# The Dentist a Teacher

**Published:** 1892-08

**Authors:** 


### Editorial, Etc.

THE DENTIST A TEACHER.—It is admitted that children's teeth do not receive the degree of attention which they demand. The public mind believes that because the period of duration of the temporary teeth is a brief one, they are not worthy the care which is bestowed upon the permanent set. Children are given little, if any, dental treatment until they are about ten years old, by which time, through the ignorance or neglect of parents almost irreparable damage has been done to the sixth-year molars, large teeth well adapted for mastication. Physicians and dentists by statistics have shown that 56 per cent. of the upper molars are decayed before the seventh year, and that the lower molars, coming first, decay at once; 20 per cent, during the first year, and 70 per cent. by the third year, after eruption.

The number of teeth lost is now so extensive that it assumes a national calamity, for the digestion being impaired the physical stamina of the nation is injured. The evils arising from insufficient mastication caused by the loss of teeth can not be exaggerated. The dental profession is, in a great measure, responsible for the ignorance respecting the mouth and the teeth. If schools would engage the services of properly-qualified dentists, the needless loss of many teeth may be prevented. Instruction in the daily care of the teeth could be given.

"To avoid the wholesale destruction of teeth, to serve their patients' best interests, and to save themselves very many difficult, tedious and wearing operations, which at best are not always entirely satisfactory to the operator, yet are the best that may be done from the condition of affairs, many operators have lately adopted the plan of *notifying their patients when they*

*should come.* This plan, although it may have the look of being mercenary, or of seeking to drum up patients, is one that suits such persons who are worth having as patients, and these are pleased to have the burden of *remembering* when to go to the dentist shifted from their shoulders. It is customary then, with many dentists, to have *two diaries*, and after having completed for the patient to put his or her name and address down in one of these, at a certain *hour* and *date*, three or six months in advance, accordingly as he thinks the patients' teeth need watching.

"At the beginning of the year these names are copied in the *new diary* and *each week* postals are sent to the different patients notifying them of the day and hour of their appointment. By this plan a clientelle is established, patients come regularly, their teeth are watched and extensive decay thwarted, the responsibility of their loss is, to a great measure shifted from their shoulders, their bills are comparatively light, since the amount of work, by such a plan, is necessarily small, there are few or no new cases, and often no severe toothache, so that when the plan is once established, it works to the satisfaction of all concerned."

That the subject is worthy of public attention is witnessed by recent articles in current literature. The one quoted in the July number of this Journal from *Harper's Weekly* concluded with these words: "Take it all in all, care of the teeth *pays*, in comfort, in beauty, in the conservation of health from youth to old age." The *Youth's Companion*, which enters so many homes in this country as a moulder and guide of conduct, also has an editorial which says:

"All diseases which profoundly affect the nutrition influence the development of the teeth; and since the growth of the teeth is mainly limited to the age of childhood, their condition is especially influenced by children's diseases.

"Faulty nutrition or severe wasting illness show themselves nowhere more prominently than in the development of the bones and teeth; and on the other hand good teeth in children play a very important part in producing a healthful and robust manhood or womanhood. Decaying and loosened teeth directly favor imperfect mastication and consequent indigestion.

"Indigestion favors poor nutrition; it causes the secretions of the mouth to become acid in reaction—a perversion of the normal reaction of the saliva, which attacks the teeth and favors their rapid decay.

"A case of this kind has lately been observed. A child, naturally good-natured but "spoiled" by indulgent parents, was allowed to eat fresh cakes, expensive candies, jams, pastries. Attacks of indigestion, with vomiting of the acid contents of the stomach, supervened. The teeth softened, "became poor" at an early age.

"In order to assure such a child a healthful bodily development and protect it from the evils of subsequent attacks of indigestion, there must be something more than a correction of its diet. The teeth should be filled. This guards against disease of the alveolar process, or the bony portion of the jaw into which the roots of the teeth are inserted, against an unsymmetrical growth of the jaw itself, and against an involvement of the second tooth, just developing beneath the first.

"A regular supervision of children's teeth would save large dentist's bills, and would undoubtedly tend to a healthier, stronger race of mankind. From the time of the first appearance of the teeth through the gums, they should be subjected to a rubbing twice a day with a soft rag and lime water, until the twelfth month of infancy, when a soft brush should be substituted.

"Frequent visits to the dentist are an absolute necessity.

"Children who are allowed to eat warm bread, rich pastries, cakes and candies are almost invariably subject to habitual attacks of indigestion. The far-reaching effects of such attacks can be avoided by the prohibition of such food. Meat, not too tender, and crusts of bread are excellent objects upon which a child's teeth may be exercised and strengthened."

